# Controlled dehydration, structural flexibility and gadolinium MRI contrast compound binding in the human plasma glycoprotein afamin

**DOI:** 10.1107/S2059798319013500

**Published:** 2019-11-19

**Authors:** Andreas Naschberger, Pauline Juyoux, Jill von Velsen, Bernhard Rupp, Matthew W. Bowler

**Affiliations:** a Department of Genetic Epidemiology, Medical University Innsbruck, Schöpfstrasse 41, A-6020 Innsbruck, Austria; b Grenoble Outstation, European Molecular Biology Laboratory, 71 Avenue des Martyrs, 38042 Grenoble, France; c C.V.M.O., k. k. Hofkristallamt, 991 Audrey Place, Vista, California, USA

**Keywords:** afamin, glycoproteins, hydrophobic ligands, conformational variability, Wnt signalling, gadoteridol, MRI contrast agents

## Abstract

Afamin, which is a human blood plasma glycoprotein, a putative multifunctional transporter of hydrophobic molecules and a marker for metabolic syndrome, poses multiple challenges for crystallographic structure determination, both practically and in analysis of the models. Several hundred crystals were analysed, and an unusual variability in cell volume and difficulty in solving the structure despite an ∼34% sequence identity with nonglycosylated human serum albumin indicated that the molecule exhibits variable and context-sensitive packing, despite the simplified glycosylation in insect cell-expressed recombinant afamin. Controlled dehydration of the crystals was able to stabilize the orthorhombic crystal form, reducing the number of molecules in the asymmetric unit from the monoclinic form and changing the conformational state of the protein. An iterative strategy using fully automatic experiments available on MASSIF-1 was used to quickly determine the optimal protocol to achieve the phase transition, which should be readily applicable to many types of sample. The study also highlights the drawback of using a single crystallographic structure model for computational modelling purposes given that the conformational state of the binding sites and the electron density in the binding site, which is likely to result from PEGs, greatly varies between models. This also holds for the analysis of nonspecific low-affinity ligands, where often a variety of fragments with similar uncertainty can be modelled, inviting interpretative bias. As a promiscuous transporter, afamin also seems to bind gadoteridol, a magnetic resonance imaging contrast compound, in at least two sites. One pair of gadoteridol molecules is located near the human albumin Sudlow site, and a second gadoteridol molecule is located at an intermolecular site in proximity to domain IA. The data from the co-crystals support modern metrics of data quality in the context of the information that can be gleaned from data sets that would be abandoned on classical measures.

## Abstract 2

Controlled dehydration experiments have revealed a new crystal form of afamin, a human blood plasma glycoprotein and transporter of hydrophobic molecules. The comparison shows substantial molecular plasticity and amplifies the necessity to examine multiple crystal forms and to refine multiple models, while at the same time the new structure cautions against the interpretation of fatty-acid ligand density in crystals derived from PEGs as major precipitants. An isomorphic low-resolution structure model suggests that afamin is capable of transporting gadoteridol (Gd-DO3A), a magnetic resonance imaging compound.

## 1. Introduction   

Afamin is a human plasma glycoprotein and a member of the albumin gene family that has been reported to be a multifunctional transporter of hydrophobic molecules such as vitamin E (Voegele *et al.*, 2002 ▸) and a potential binding partner for Wnt signalling proteins (Mihara *et al.*, 2016 ▸). High afamin concentrations in human plasma are associated with all major parameters for metabolic syndrome, such as high blood glucose, as well as dyslipemia, obesity and high blood pressure, and also pre-eclampsia and ovarian cancer (Dieplinger *et al.*, 2009 ▸; Kronenberg *et al.*, 2014 ▸; Tramontana *et al.*, 2018 ▸). A potential role of afamin in glucose metabolism in papillary thyroid carcinoma has been reported (Shen *et al.*, 2016 ▸). Afamin was shown *in vitro* to form a 1:1 complex with most of the 19 Wnt proteins (Mihara *et al.*, 2016 ▸), maintaining bio­logical activity for Wnt3 and Wnt3a and partial activity for Wnt1, Wnt7a, Wnt7b, Wnt8a, Wnt9b and Wnt10b, which play a crucial role in cell differentiation during embryogenesis and are involved in the development of various diseases, including cancer (Nusse & Varmus, 2012 ▸; Nile & Hannoush, 2019 ▸). A compelling computational model of lipid-bound afamin in a monoclinic crystal form (PDB entry 5okl) docked with a homology model of Wnt3a indicated that afamin can capture the acyl chain of the palmitoylated Ser209 of Wnt3a in a deep hydrophobic pocket (Naschberger *et al.*, 2017 ▸).

The preparation and crystallization of afamin has posed significant challenges (Altamirano *et al.*, 2018 ▸). In contrast to human serum albumin (hSA), which is exported from the liver as a nonglycosylated chain, human afamin is highly and variably enzymatically glycosylated *in vivo* (Jerkovic *et al.*, 2005 ▸). Neither complex glycosylation nor the possible nonspecific binding of lipid components from the expression media bode well for crystallization. In our hands, only afamin purified from the Sf21 baculoviral system, as described in Section 2.1, yielded crystals, and only a few data sets could be successfully processed from several hundred mounted crystals (Naschberger *et al.*, 2017 ▸), revealing a large variability in unit-cell dimensions and symmetry.

Crystals can sometimes be improved by dehydration. A reduction of the mole fraction of water surrounding crystals of macromolecules, either by changing the components of the mother liquor or using specific humidity-control devices, can induce phase transitions (Heras & Martin, 2005 ▸; Newman, 2006 ▸; Russo Krauss *et al.*, 2012 ▸). These transitions can lead to an increase in the order within the crystal lattice, resulting in increased diffraction quality or other changes such as an increase in symmetry or reduced diffraction anisotropy (Bowler *et al.*, 2006 ▸; Cramer *et al.*, 2000 ▸; Hu *et al.*, 2011 ▸; Kadlec *et al.*, 2011 ▸; Raj *et al.*, 2017 ▸; Zerrad *et al.*, 2010 ▸; Scherer *et al.*, 2014 ▸). There are many examples of spectacular increases in diffraction quality, but often small changes can have similarly decisive beneficial results. Here, we describe how the controlled dehydration of afamin crystals was able to stabilize a new orthorhombic crystal form, increasing the resolution, inducing a change in symmetry and increasing the number of intermolecular contacts, and leading to a more complete model. An iterative process using automated protocols on the ESRF/EMBL beamline MASSIF-1 (Svensson *et al.*, 2015 ▸) to combine running automated dehydration experiments (Bowler, Mueller *et al.*, 2015 ▸) with multi-crystal data collection (Svensson *et al.*, 2018 ▸) allowed the rapid determination of optimized conditions and selection of the best data set.

**Figure 1:**
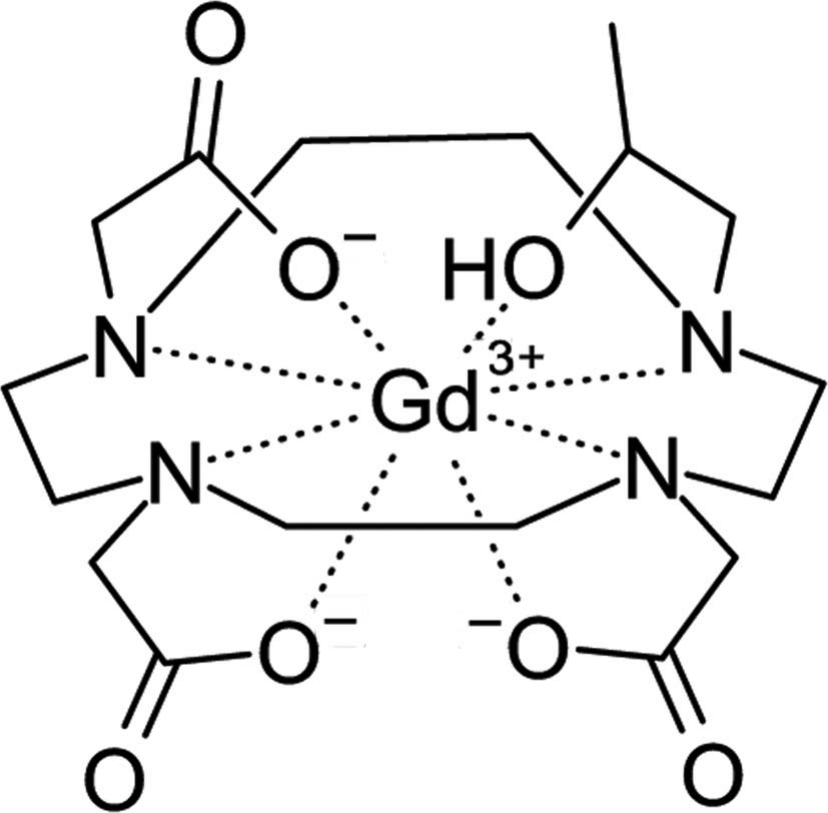
The structure of the paramagnetic MRI contrast agent gadoteridol (Gd-DO3A).

Human serum albumin is a promiscuous drug transporter that is known to transport paramagnetic magnetic resonance imaging (MRI) contrast-enhancement agents (Fasano *et al.*, 2005 ▸). The structural similarity of afamin to hSA (Naschberger *et al.*, 2017 ▸) suggests that afamin might also be able to transport cargo in the bloodstream. Tissue-specific transport by afamin could greatly enhance the resolution of MRI imaging in blood-flow studies (see, for example, Schultz *et al.*, 1999 ▸). As no crystal structure of albumin with a contrast agent is yet available, we selected gadoteridol (Gd-DO3A; Fig. 1 ▸), which is used in medical applications as an MRI contrast agent (Caravan, 2009 ▸) and in crystallography as a lanthanide phasing compound (Girard *et al.*, 2003 ▸). We were able to identify likely Gd-binding sites in a similar orthorhombic, low-resolution electron-density map of afamin by co-crystallization with Gd-DO3A. In view of the marginal quality of the data and the incomplete model, the details of Gd-DO3A binding remain tentative.

The availability of four independent afamin structure models from two different crystal forms provides insight into the flexibility and dynamics of the molecule, which point towards a significant conformational adaptability when binding to hydrophobic molecules, metal chelates or proteins with hydrophobic acylation such as Wnt, supporting the potential function of afamin as a promiscuous transport molecule in human plasma. The palmitoleic acid present in Wnt proteins and identified by lipid analysis in afamin could plausibly be modelled into electron density in a deep central hydrophobic cavity that is present only when the molecule adopts the most open conformation (Naschberger *et al.*, 2017 ▸). As modern structural biology beamlines now allow the collection of vast amounts of data, the option of refining multiple models should not be ignored.

## 2. Experimental details   

### 2.1. Protein expression and purification   

The preparation of afamin (UniProt entry P43652, AFAM_HUMAN) has been described previously (Altamirano *et al.*, 2018 ▸) and the expression of afamin in the *Spodoptera frugiperda* 21 (Sf21) insect-cell line and the subsequent purification of the glycoprotein for structural studies have also been reported (Naschberger *et al.*, 2017 ▸).

### 2.2. Crystallization   

#### 2.2.1. Native afamin crystals   

Initial crystals were obtained at 291 K via the sitting-drop method in a 96-well plate format (SwissSci) set up with a Phoenix robot (Art Robbins Instruments, Sunnyvale, California, USA) using the PEG Wizard Screen (30% PEG 1000, 200 m*M* ammonium acetate). The crystals were optimized using streak-seeding, while 6-amino­hexanoic acid (ACA) was added to a final concentration of 3%(*w*/*v*) in a hanging-drop vapour-diffusion setup (VDX plate; Hampton Research). 1.5 µl droplets of afamin stock solution (5 mg ml^−1^, purified by size-exclusion chromatography) were mixed with 1.5 µl reagent (30% PEG 1000, 170 m*M* ammonium acetate, 3% ACA) followed by micro-seeding immediately after pipetting with no pre-equilibration of the drop. The actual crystallization drop pH of 6.5 was determined by measurement. Different seed dilutions were obtained by loading a cat whisker once with crushed crystals followed by sequential streak-seeding into six different drops.

#### 2.2.2. Afamin–Gd-DO3A co-crystals   

To obtain crystals of afamin containing gadoteridol (Gd-DO3A), the protein was incubated with 10 m*M* Gd-DO3A (NatXRay, Grenoble, France) and immediately sent for high-throughput screening at the HTX laboratory at EMBL, Grenoble, France. Initial crystallization conditions were obtained as 25% PEG 4000, 0.2 *M* ammonium sulfate, 0.1 *M* sodium acetate pH 4.6. These conditions were refined, with crystals appearing in 23–28% PEG 4000, 0.2 *M* ammonium sulfate, 0.1 *M* sodium acetate pH 5.5. All crystals were harvested by laser photoablation and cryocooled using the CrystalDirect robot (Zander *et al.*, 2016 ▸; Pellegrini *et al.*, 2011 ▸).

### 2.3. Data collection   

**Figure 2:**
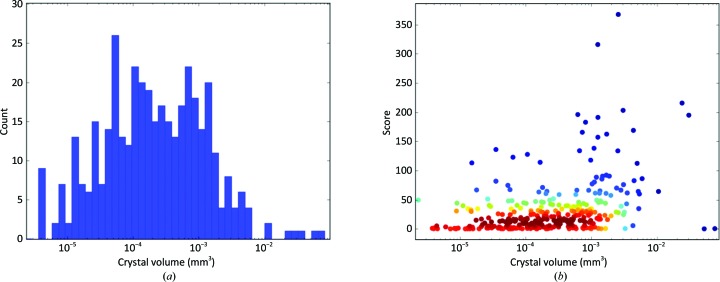
Volume distribution of crystals of afamin. (*a*) Distribution of volumes observed for afamin crystals and (*b*) the crystal volume against the *Dozor* score (a measure of quality based on the radial intensity over background noise). The plot demonstrates that the small number of crystals obtained that diffract to high resolution is not related to the volume, requiring large-scale screening.

Prior to dehydration experiments, 348 crystals were screened (Fig. 2 ▸) on the fully autonomous ESRF beamline MASSIF-1 (Bowler, Nurizzo *et al.*, 2015 ▸; Nurizzo *et al.*, 2016 ▸) at a fixed wavelength of 0.966 Å. Data were processed by the *EDNA* automated data-processing pipeline (Monaco *et al.*, 2013 ▸) employing *XDS* and *XSCALE* (Kabsch, 2010 ▸) and *POINTLESS*/*AIMLESS*/*CTRUNCATE* from the *CCP*4 suite (Winn *et al.*, 2011 ▸) and *phenix.xtriage* from the *Phenix* suite (Adams *et al.*, 2011 ▸). Strategy calculations accounted for flux and crystal volume in the data-collection parameter prediction for complete data sets, and in the case of the monoclinic *mP* lattice the symmetry was pre-set (Svensson *et al.*, 2015 ▸). Analysis of the parameters of all of the afamin crystals tested (Svensson *et al.*, 2019 ▸) shows that large-scale screening was required as a wide variety of crystal volumes were observed, with very few diffracting to high resolution (Fig. 2 ▸).

It rapidly became apparent that widely varying unit-cell parameters, leading to a variation in unit-cell volumes of as much as 30–35%, were present in a primitive orthorhombic crystal form (*oP* lattice), in addition to several instances of a related primitive monoclinic cell (*mP*). While we were able to refine the monoclinic form (space group No. 4, *P*2_1_) with two molecules in the asymmetric cell as detailed previously (Naschberger *et al.*, 2017 ▸), the merging statistics for the orthorhombic crystal forms (largely space group No. 18, *P*2_1_2_1_2) were generally poor. Despite nominal resolutions of around 2 Å or better, the refinement stalled at high *R* values without obvious avenues for improvement of the ortho­rhombic structure model. Dehydration experiments were therefore conducted on about 100 orthorhombic crystals to determine whether this form could be stabilized.

Afamin–gadoteridol complex crystals were screened on MASSIF-1 using automatic protocols for the location and the optimal centring of crystals (Svensson *et al.*, 2015 ▸). The beam diameter was selected automatically to match the best crystal volume (Svensson *et al.*, 2018 ▸). Strategy calculations accounted for flux and crystal volume in the data-parameter prediction for complete data sets. Despite appearing from different precipitant conditions, the screening of 164 poorly diffracting crystals yielded highly anisotropic *oP* diffraction data similar to the previously obtained orthorhombic crystal form but with a significantly smaller unit cell. After pre-processing with *XDS* the data were submitted to the *STARANISO* server (Tickle *et al.*, 2018 ▸) and the *R*_free_ reflections from dehydrated orthorhombic afamin (PDB entry 6fak) were used for all orthorhombic data sets. Despite poor merging statistics and low completeness (Table 1 ▸), the anisotropy-corrected data gave clear molecular-replacement solutions in space group No. 18, *P*2_1_2_1_2, but with PDB entry 5okl chain *A* from monoclinic afamin as a template (Naschberger *et al.*, 2017 ▸) using *Phaser* (McCoy *et al.*, 2007 ▸). Although no significant anomalous signal could be extracted from the data recorded at 12.835 keV (above the Gd *L*_III_ edge of 7.243 keV), strong positive difference peaks indicated the likely presence of three Gd sites, of which two were in a pair exhibiting the same Gd–Gd distance as observed in high-resolution models of lysozyme complexed with Gd-DO3A (Gorel *et al.*, 2017 ▸; Holton *et al.*, 2014 ▸; Girard *et al.*, 2002 ▸). Partial model building with *Coot* (Emsley *et al.*, 2010 ▸) and refinement with *REFMAC* (Murshudov *et al.*, 2011 ▸) proceeded as described for the monoclinic form of afamin (Naschberger *et al.*, 2017 ▸). As a consequence of the high data anisotropy, poor merging statistics and low completeness, various disordered regions could not be modelled owing to streaky and discontinuous maps. While the core regions of afamin were well defined and had good geometry, the *R*_free_ values never decreased below 33% and only an incomplete model could be obtained.

### 2.4. Dehydration experiments   

**Figure 3:**
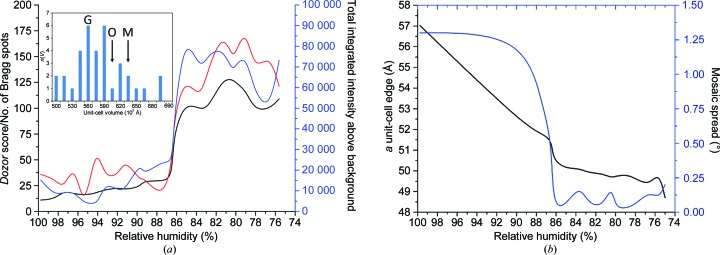
The effect of dehydration on the properties of afamin crystals. (*a*) The increase in diffraction quality upon dehydration of an afamin crystal. The measures of quality used are the number of Bragg spots (black), the total integrated intensity above background (blue) and the *Dozor* score, which is based on the radial intensity over background noise (red). The diffraction-quality indicators increase dramatically around 86% relative humidity (RH). The inset shows a histogram of the unit-cell volumes of the afamin crystal forms (M, monoclinic; O, orthorhombic dehydrated; G, Gd-DO3A–afamin complex). (*b*) The decrease in the *a* cell dimension and the mosaic spread on decreasing the RH.

**Figure 4:**
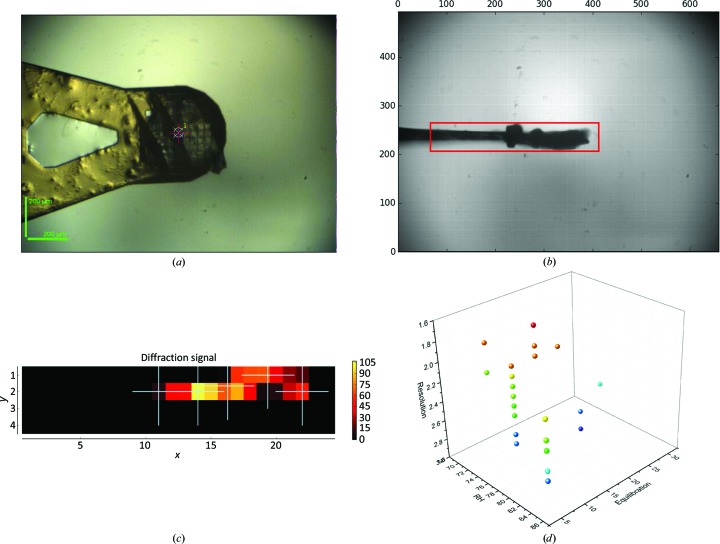
Multi-crystal strategy for optimizing the dehydration protocol. (*a*) Multiple crystals were loaded onto micro-mesh mounts, subjected to various dehydration protocols and cryocooled directly. (*b*, *c*) Multi-crystal data collection was then run on the supports on MASSIF-1, allowing the dehydration protocols to be assessed despite crystal variation. (*d*) Finally, the results could be assessed, showing which protocol should be used and applied to further crystals.

Dehydration experiments were performed using an HC-Lab instrument (Arinax, France; Sanchez-Weatherby *et al.*, 2009 ▸). The relative humidity (RH) in equilibrium with the mother liquor was predicted to be 98% (Bowler *et al.*, 2017 ▸; Wheeler *et al.*, 2012 ▸) and single crystals were mounted at this RH on micromesh mounts (MiTeGen, Ithica, USA) on the RoboDiff goniometer (Nurizzo *et al.*, 2016 ▸) at the ESRF beamline MASSIF-1 (Bowler, Nurizzo *et al.*, 2015 ▸). An automated workflow was then launched (Bowler, Mueller *et al.*, 2015 ▸) that decreased the RH in steps of 1% with an equilibration time of 5 min, with data collection and analysis at the end of each step. When the RH reached 85% a significant increase in diffraction quality was observed (Fig. 3 ▸ *a*, Supplementary Movie S1) accompanied by a reduction in the *a* cell edge and the mosaic spread (Fig. 3 ▸ *b*). The resolution increase was maintained until a RH of 75% was reached, whereupon diffraction was lost (Supplementary Movie S1). This transition was confirmed by repeating the protocol on five crystals in the same manner. In order to optimize this transition, a number of protocols were attempted that also incorporated automated data-collection procedures (Svensson *et al.*, 2015 ▸). A major bottleneck in refining the best protocol was the need to screen crystals before starting dehydration; therefore, in order to cover a wide range of conditions a multi-crystal protocol was used. Multiple *oP*-form crystals (3–7) were mounted on mesh loops (Figs. 4 ▸ *a* and 4 ▸ *b*) and subjected to dehydration gradients to 80, 75 and 70% RH in either multiple or single steps, also varying the final equilibration times (5–20 min). Once the protocols were completed, the crystals were then cryocooled directly (Pellegrini *et al.*, 2011 ▸) and launched for autonomous characterization and data collection using the software and procedures (Figs. 4 ▸ *b* and 4 ▸ *c*) described in Section 2.3 for multiple crystals (Svensson *et al.*, 2018 ▸). The obtained data sets were then analysed for quality and this information was fed back into subsequent rounds of dehydration with a refined protocol (Fig. 4 ▸ *d*). The final protocol used was to dehydrate crystals to an RH of 75% in a single step with a final equilibration time of 15 min. Three rounds of optimization were performed using a total of 134 crystals. Using this procedure, the orthorhombic crystal form could be stabilized and data were collected that extended to a Bragg spacing of ∼1.8 Å. The best data were manually reprocessed using the software described in Section 2.3 with the statistics provided in Table 1 ▸. No twinning or pseudosymmetry (translational NCS) were detected in the data sets used for the dehydrated model refinement, and the modest anisotropy in the orthorhombic data was accounted for in anisotropic scaling (Murshudov *et al.*, 2011 ▸) during refinement.

### 2.5. Multi-conformer refinement   

Given the highly dynamic nature of the afamin molecule, time-dependent molecular-dynamics multi-conformer refinement as implemented in *Phenix* (Burnley *et al.*, 2012 ▸) was conducted. These models are snapshots of conformations during data-restrained molecular-dynamics refinement and represent a convolution of molecular disorder and model degeneracy. They were generated for the visualization and emphasis of the extent of molecular flexibility and no coordinate ensembles were deposited.

## 3. Results and discussion   

### 3.1. Effect of dehydration on crystal packing   

**Figure 5:**
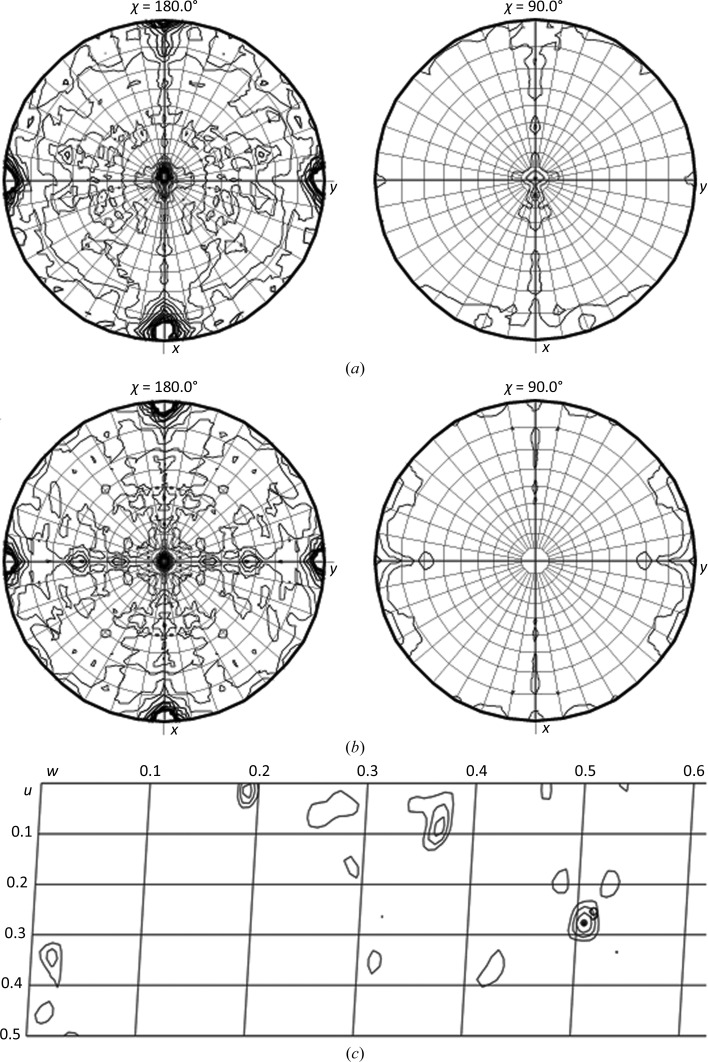
Self-rotation functions and a native Patterson map of the monoclinic and orthorhombic crystal forms show the close relation between NCS and CS. The self-rotation function shows that the peaks from the noncrystallographic symmetry in the monoclinic form (*a*) are very close to those from the crystallographic symmetry in the orthorhombic crystal (*b*), implying that only a small shift is needed to satisfy the requirements for higher symmetry. While the orthorhombic map (*b*) displays almost perfect *mm* symmetry, reduced symmetry along *y* in (*a*) becomes visible. The Harker section (*u*, ½, *w*) of the native Patterson map (*c*) shows a weak peak at *u*, *v*, *w* = (0.277, ½, 0.507) indicating the location of the 3.7° tilted NCS axis originating from the true crystallographic axis at *u*, *v*, *w* = (¼, ½, ½).

**Figure 6:**
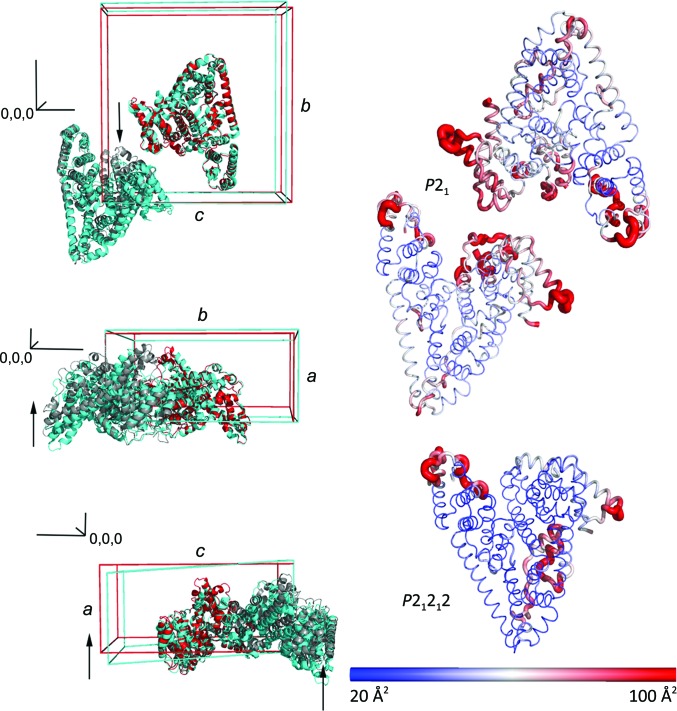
Packing and flexibility changes induced by dehydration. Left column: dehydration of the monoclinic cell (cyan) leads to a decrease in unit-cell volume and a reduction of the β angle to 90°; the latter has the most profound effect, moving one molecule in the asymmetric unit by up to 10 Å. This movement reduces the asymmetric unit to a single molecule in an orthorhombic form (red; symmetry-related mate in grey) and results in the stabilization of several loop regions in which electron density was not visible previously. Note that the axis labels refer to the monoclinic cell. In order to demonstrate the shift in unit cells, the orthorhombic form was re-indexed to match the monoclinic convention. Therefore, for the orthorhombic cell the conversion is *a*→*c* and *c*→*a*. Right column: cartoon representations of the asymmetric units of the monoclinic versus orthorhombic crystal forms. The C^α^ chains are shown coloured by *B* factor, indicating a reduction in flexibility in multiple regions.

The change between the monoclinic and orthorhombic cells is subtle. The twofold NCS axis in the monoclinic form is very close to the crystallographic twofold axis in the orthorhombic form, as illustrated by comparing the self-rotation function between the two forms and the appearance of an NCS peak in the native Patterson map (Fig. 5 ▸). The symmetry gain towards the orthorhombic form may be energetically favourable, but many of the crystals tested may not have completed the transition, leading to heterogeneity. This may be owing to slow or incomplete dehydration in the native crystallization drops or owing to packing defects as the crystals grow. The use of a humidity-control device has allowed the orthorhombic crystal form to be stabilized. The transformation involves a contraction in the *a* cell edge of over 10 Å and of the β angle by ∼3° (Fig. 6 ▸, Supplementary Movie S2). This change shifts one molecule in the asymmetric unit, allowing it to become related by crystallographic symmetry to its mate, reducing the asymmetric unit to a single molecule in the orthorhombic cell (Fig. 6 ▸). This movement also stabilizes certain loop regions that were not visible and could not be modelled in the monoclinic structures. The number of crystal contacts between symmetry-related molecules of <4 Å increases from 1999 to 2752 and the buried surface area increases from 968 to 1128 Å^2^. The result is a significant stabilization of the molecule that can be seen in the *B* factors (Fig. 6 ▸). This transition from an *mP* to an *oP* lattice takes place in the same crystal, in contrast to the formation of the smaller orthorhombic afamin–Gd-DO3A complex crystals, which were grown with a slightly different precipitant and at a different pH value (6.5 versus 4.6).

**Figure 7:**
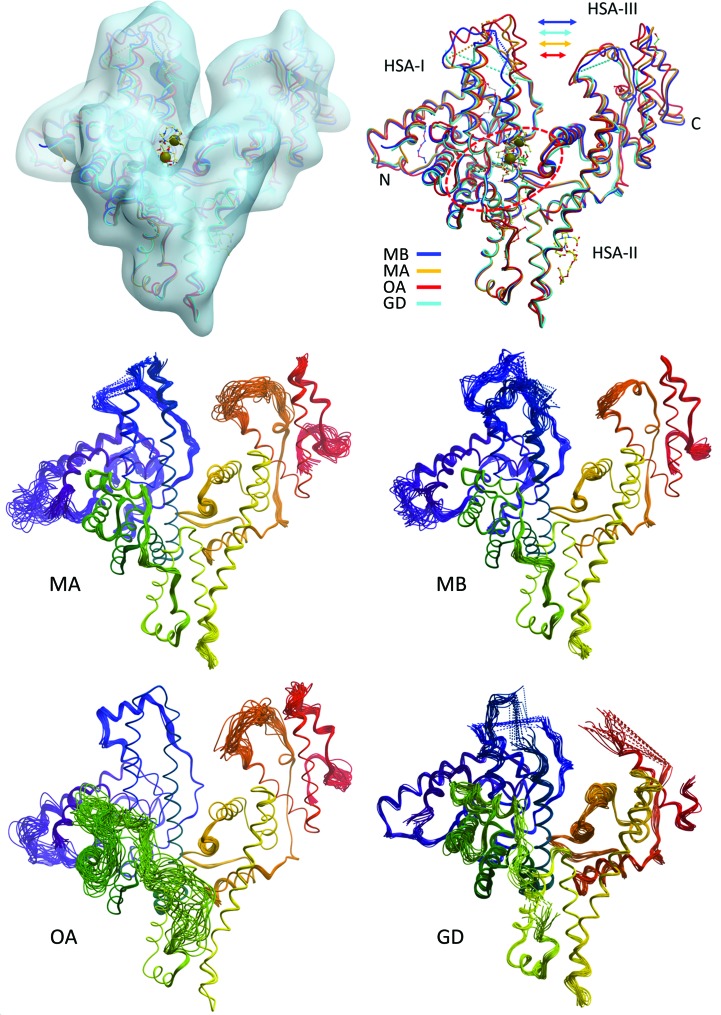
Overview of the afamin structure models. Top left: the major binding site of afamin, corresponding to the Sudlow S1 drug-binding site in albumin, is located in the centre of the heart-shaped molecule between the equivalents of hSA domains I (HSA-I) and III (HSA-III). The entrance to the deep hydrophobic cleft harbours the Gd-DO3A chelate molecules, indicated by grey spheres. Top right: the four structure models of afamin [the monoclinic model MA (PDB entry 5okl chain *A*), the monoclinic model MB (PDB entry 5okl chain *B*), the dehydrated orthorhombic model OA (PDB entry 6fak chain *A*) and the orthorhombic form of the Gd-DO3A complex GD] are superimposed on hSA domain II (HSA-II). The motion of domains I and III towards each other affects access to the binding site. The first hexose molecules of the paucimannose basic glycosylations and the PEG molecules of the dehydrated model PDB entry 6fak chain *A* are also shown as ball-and-stick models. Bottom four panels: multi-conformer refinement trajectories of the four independent afamin models, illustrating the high degree of plasticity of the afamin molecule. The backbone trace is coloured from the N-terminus (blue) to the C-terminus (red). It is noticeable that in the four structure models from related crystal forms it is not always the same parts that are disordered and missing. Recognizable examples are the green connecting helix being well defined in MA but severely disordered in the high-resolution OA, while the N-terminal region (blue) is well ordered in OA but is disordered in MB. The higher overall disorder of GD and the absence of large parts of the C-terminus (red) in GD are also distinctive. Figures were produced with *ICM-Pro* from Molsoft (Abagyan *et al.*, 2006 ▸).

The cell-volume distribution of the data sets that could be indexed (Fig. 3 ▸ *a*, inset) shows that as expected the volume shrinks by about 3.5% on dehydration. An even more dramatic cell contraction dominated by a further 6 Å contraction in *a* compared with the isomorphous ortho­rhombic dehydrated crystals and an almost 13% decrease from the monoclinic cell volume occurs in the orthorhombic afamin–Gd-DO3A complex crystals. Whether this decrease in cell volume is mediated by the single Gd-DO3A molecule located at a crystal contact (Fig. 7 ▸) or is a result of shifting local charge distributions owing to the lower crystallization pH of 5.5 versus 6.5 for native afamin remains unknown.

### 3.2. Contextual flexibility affects binding-site analysis   

**Figure 8:**
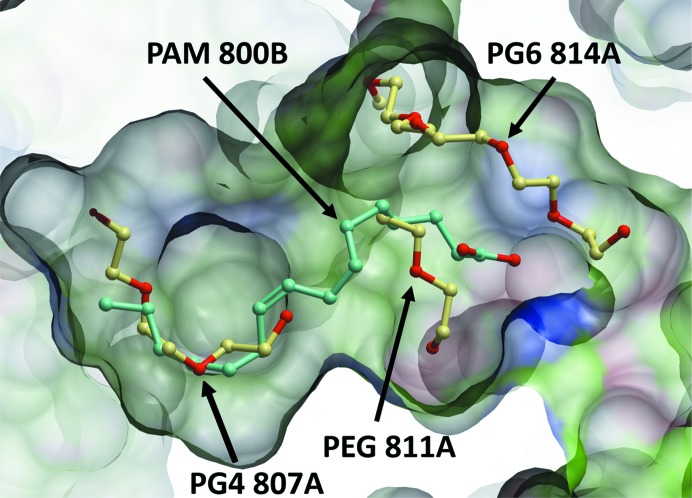
Detailed view of the hydrophobic binding cleft in the dehydrated crystal structure of afamin. A cross-section of the deep hydrophobic binding pocket (left side) is shown over transparent property-coloured residue surfaces (white, lipophilic; green, aromatic lipophilic; red, hydrogen-bonding acceptor potential; blue, hydrogen-bond donor potential). The palmitoleic acid as modelled into the cavity of PDB entry 5okl chain *B* (MB) is overlaid as a green ball-and-stick model. The PEG fragments modelled into the density of the dehydrated structure PDB entry 6fak (OA) are shown in a yellow ball-and-stick representation. A narrow second channel (top middle of the figure) is visible harbouring a long PEG fragment in PDB entry 6fak. Environment and electron density for the PEG fragments can be readily inspected via the PDB tools, for example https://www.ebi.ac.uk/pdbe/entry/pdb/6fak/bound/PG6. This figure was produced with *ICM-Pro* from Molsoft (Abagyan *et al.*, 2006 ▸).

One of the most interesting insights gained from the comparison of the four different afamin structure models, the monoclinic model MA (PDB entry 5okl chain *A*), the monoclinic model MB (PDB entry 5okl chain *B*), the dehydrated orthorhombic model OA (PDB entry 6fak chain *A*) and the orthorhombic Gd-DO3A complex GD (see Section 3.4), is that the relative motions of the domains forming the deep cleft in the centre of the heart-shaped molecule (following the hSA description) do affect the shape of the deep hydrophobic binding pocket at the equivalent of the Sudlow 1 (S1) drug-binding site in hSA (Naschberger *et al.*, 2017 ▸). The breathing motions that occur in the transition from the more open MB conformation to the tighter MA and OA conformations (Fig. 7 ▸) also affect the S1 binding site. In the dehydrated OA form an additional minor pocket opens up, extending the primary S1 binding cleft, which is then occupied by what we believe to be a PEG fragment (see also Section 3.3; Fig. 8 ▸).

While one can argue that the OA binding site of the dehydrated crystal structure does not represent a native solution conformation, Fig. 7 ▸ does amplify the concerns that a single-crystal structure, particularly in a restrictive crystal packing, may not be sufficient as a basis for a drug-lead discovery study (Dym *et al.*, 2016 ▸). The power of multiple crystal forms to explore the conformational space of a protein in its native solvent environment has repeatedly been made (see, for example, Naschberger *et al.*, 2016 ▸). Given modern high-throughput crystallography methods, as many different crystal forms should be examined as possible in order to obtain a complete picture, particularly in the case of highly promiscuous small-molecule and drug transporters, as exemplified by human albumin and afamin. The frequent presence of PEG molecules in crystallization cocktails adds an additional level of uncertainty to structure-guided drug-lead discovery (Dym *et al.*, 2016 ▸), in particular in the case of fatty-acid chains.

### 3.3. Probing the binding site for hydrophobic molecules: different crystal forms can lead to different ligand occupancies   

The most prominent feature of the afamin molecule is the deep, central binding cavity that extends almost across the entire molecule (Fig. 8 ▸). While the solvent-exposed region of this deep cavity (in which the Gd-DO3A is located) is in the vicinity of the Sudlow 1 drug-binding site in hSA, the inner lining of the much deeper pocket in afamin is almost exclusively formed by hydrophobic and lipophilic residues. This deep hydrophobic pocket is likely to be the key anchor for the interaction of palmitoylated Wnt3a with afamin (Mihara *et al.*, 2016 ▸), and distinct electron density in this cavity of the non­dehydrated monoclinic model chain *B* was attributed to palmitoleic acid (PAM) and was supported by lipid analysis of the purified and crystallized afamin sample (Naschberger *et al.*, 2017 ▸). Additional supporting evidence for fatty-acid binding such as lipid analysis and a careful analysis of the chemical site environment is almost always necessary when PEGs are a component of the crystallization cocktail, because at common resolutions (around 2.5–2.0 Å) the electron-density shape alone does not allow a clear distinction between an aliphatic fatty-acid tail and a PEG molecule.

The possibility of forcing PEG molecules into binding sites as surrogate probes by dehydration is intriguing. In the example of afamin, the presence of the secondary binding channel occupied by a PEG molecule (Fig. 8 ▸) opens up the speculation that afamin could, at least from a structural point of view, also accommodate moieties with two fatty-acid chains, such as phosphatidylcholines.

### 3.4. Gd-DO3A in afamin: the case for salvaging poor data and how to disseminate them   

Judging by historically used criteria such as a high-resolution data cutoff at an 〈*I*/σ(*I*)〉 of 2, the Gd data should not be useful beyond ∼3.4 Å resolution. Beyond this resolution the data are highly anisotropic and the completeness is unacceptably low. As expected from these statistics, we were unable to extract any anomalous difference signal, which is expected to be 3.4% at 12.835 keV (0.966 Å) and thus detectable with reasonable data quality (Lemke *et al.*, 2002 ▸) and full occupancy given the Gd *L*_III_ edge of 7.243 keV. In the absence of anomalous signal and in view of the metrics, the data would have been discarded by default. Nonetheless, we were able to extract useful information from these data.

**Figure 9:**
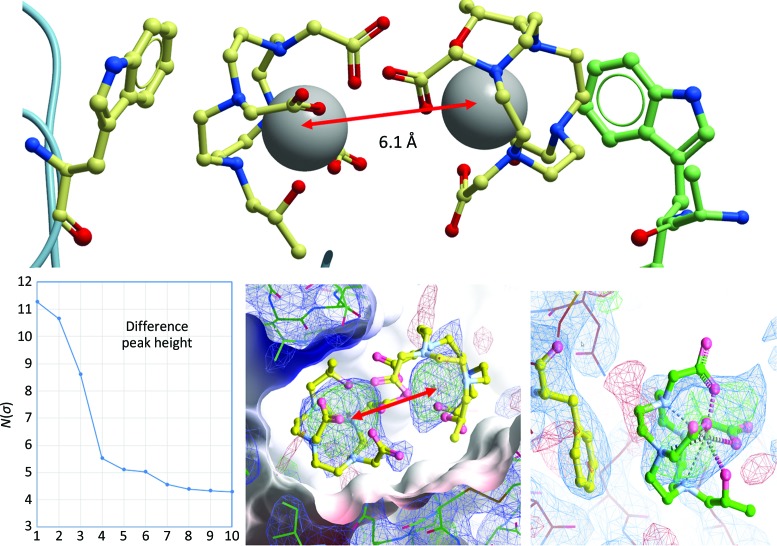
Gd-DO3A sites in lysozyme and afamin. The top panel shows the arrangement of the Gd-DO3A sites in a high-resolution model of lysozyme (PDB entry 1h87; Girard *et al.*, 2003 ▸), in which the Gd pair forms a crystal contact involving hydrophobic interaction of the DO3A methyl groups with Trp62 of one molecule and Trp123 of a symmetry mate. A similar arrangement of the Gd ions exists in afamin, where two of the strongest difference peaks (inset) appear in the central hydrophobic binding cleft. The placed Gd atoms refined with an occupancy of between 0.9 and 0.7, and the distance between them is also 6.1 Å, as in the lysozyme model, with sufficient space in the binding site to accommodate the DO3A crowns of the Gd-DO3A complexes. The bottom centre panel shows *mF*_o_ − *DF*_c_ positive OMIT difference electron density displayed at a 2.5σ level in green and 2*mF*_o_ − *DF*_c_ density displayed at 0.8σ in blue. The DO3A crowns are not refined but are placed in an orientation corresponding to that in PDB entry 1h87. The methyl groups of the placed DO3A crowns face towards a phenylalanine lining the bottom (left side of the figure) of the binding pocket, resembling the interactions observed in PDB entry 1h87. A similar interaction can be proposed for the third Gd site located at a crystal contact in afamin (bottom right). The top figure was produced with *ICM-Pro* from Molsoft (Abagyan *et al.*, 2006 ▸) and the electron-density figures were produced with *Coot* (Emsley *et al.*, 2010 ▸).

Molecular replacement with *Phaser* (McCoy *et al.*, 2007 ▸) yielded the same solutions irrespective of whether the data to 2.7 Å resolution were corrected for anisotropy with *STARANISO* (Tickle *et al.*, 2018 ▸) or the internal anisotropy correction in *Phaser* was applied. The log-likelihood gain and final TFZ scores were identical within 5% regardless of which anisotropy correction was applied, but were distinctly lower without any anisotropy correction. The success of MR even in the absence of anisotropy correction is not surprising because the molecular envelope is largely determined by low-resolution reflections, which were less affected by anisotropy and completeness. The best solution was obtained with the monoclinic afamin search model MA (PDB entry 5okl chain *A*, TFZ 27.9, *R*_free_ 0.48), while the worst solution was obtained with the dehydrated ortho­rhombic afamin model OA (PDB entry 6fak chain *A*, TFZ 22.5, *R*_free_ 0.52). The reason might be that the domain arrangements of hSA domains I and III (Fig. 7 ▸) in the MA and GD models are similar. Immediately after MR, three positive difference electron-density peaks became prominent (Fig. 9 ▸), while smaller difference electron-density peaks were mostly located in unmodelled or incorrectly modelled regions of the map.

After a few rounds of rebuilding it became clear that large parts of the protein had become severely disordered in the Gd-DO3A co-crystals, and owing to streaking and discontinuity in many parts of the map the model could not be completed. Refinement of the model stalled at *R*_free_ values of ∼0.36 despite good geometry for the traceable part of the model. At this point, Gd atoms were placed with occupancy set to 0.7 so that the *B* factors refined to values of about 150% of the mean environmental *B* factor. Subsequent occupancy refinement indicated Gd occupancies of between 0.9 and 0.7. The protrusions in the large density blobs and difference density suggested additional features around the Gd atom, but we were unable to determine and refine the orientation of the DO3A ‘crown’ around the Gd-DO3A. However, the refined distance of 6.1 Å between the two Gd peaks corresponds exactly to the distance observed in the high-resolution lysozyme structures, and we placed, but did not refine, the DO3A crowns in the two poses determined in PDB entry 1h87 (Girard *et al.*, 2003 ▸). In these poses there were no collisions, and a possible interaction of the eight methyl groups of the DO3A crown with an aromatic residue lining the bottom of the binding pocket is similar to that observed in lysozyme. The same holds for the third Gd-DO3A located at an afamin crystal contact (Fig. 9 ▸).

## 4. A case for depositing anisotropic models: but how?   

Our proposition of Gd-DO3A being present in afamin is based on a preponderance of evidence in the form of plausibility arguments, augmenting the reasonable evidence for strong Gd difference density. This is certainly far from proof beyond reasonable doubt (which anomalous methods would have provided), but is plausible enough to suggest further studies, particularly regarding the structural basis of transport of contrast agents by albumin (Caravan, 2009 ▸) and possible differences in the tissue distribution of albumin versus afamin (Kronenberg *et al.*, 2014 ▸). We therefore decided to submit the incomplete and anisotropic model to the PDB, despite the poor data-processing and refinement statistics. The original diffraction images for all structures described here, in line with recent IUCr policy developments (Helliwell *et al.*, 2019 ▸), are available for download from the ESRF data portal (Naschberger *et al.*, 2018*a* ▸,*b* ▸, 2019 ▸).

While our modest claim of Gd being present is reasonably plausible and of general interest, a valid question is whether the deposition of an incomplete model, and one lacking in details of the binding site, would perhaps contaminate the PDB and likely invite the ire of rote statistics data-miners, a problem that we have cautioned against repeatedly (Wlodawer *et al.*, 2018 ▸; Weichenberger *et al.*, 2017 ▸). This concern largely originates from the fact that descriptors for highly anisotropic data and models cannot adequately be deposited with the PDB, and thus lead to misleading statistics. With the increasing success of anisotropy-correction methods and the corresponding servers (Strong *et al.*, 2006 ▸; Tickle *et al.*, 2018 ▸), the limitations of isotropic, scalar presentation of diffraction data metrics are inadequate and outdated (Rupp, 2018 ▸). Our Gd model deposition illustrates the case.

The validation report for the Gd model states an automatically extracted resolution limit of 2.69 Å. This is misleading given the anisotropy and the correspondingly low completeness (11% spherical in the highest resolution bin; Table 1 ▸) of the data. User expectations of model quality based on an isotropic model resolution of 2.69 Å cannot be met, and neither can extrapolations of its usability for other purposes than the support of the presence of Gd. As a minimum, there needs to be a means of depositing, or preferably extracting from the respective anisotropy server logs or unmerged original diffraction data, elliptic resolution and completeness limits. Suggestions for data presentation and the annotation of consequences for the overall limitations of model anisotropy have been made (Rupp, 2018 ▸). The anisotropy reported in Section 4 of the validation report is likely to be overlooked and is also based on the submission of already elliptically corrected data and thus is not sufficient.

What is noteworthy is that in contrast to high merging *R* values precluding any acceptance of a structure model based on the Table 1 ▸ statistics, CC_1/2_ seems to be a robust measure indicating that such data in principle can be used to extract at least some useful and valid information. Finally, it would be useful to allow annotation by the depositor of unusual features, restrictions or warnings of shortcoming at the time of model deposition. A clear reference to such user-defined limitations, akin to the caveat statements, should be a prominent and data-minable element in the final deposition.

## Supplementary Material

PDB reference: afamin, 6fak

PDB reference: complex with Gd-DO3A, 6rq7

Supplementary Movie S1. DOI: 10.1107/S2059798319013500/di5032sup1.wmv

Supplementary Movie S2. DOI: 10.1107/S2059798319013500/di5032sup2.mp4

Raw diffraction images for the monoclinic (non-dehydrated) crystal form of afamin: https://doi.esrf.fr/10.15151/ESRF-DC-142893590

Raw diffraction images of the orthorhombic dehydrated crystal form of afamin: https://doi.esrf.fr/10.15151/ESRF-DC-142915526

Raw diffraction images for afamin with gadoteridol: https://doi.esrf.fr/10.15151/ESRF-DC-186857652

## Figures and Tables

**Table 1:** Crystallization, data-collection and refinement statistics for afamin structure models Values in parentheses are for the highest resolution shell.

Model	Chains *A* and *B* in PDB entry 5okl (MA, MB)	Chain *A* in PDB entry 6fak (OA)	Chain *A* in PDB entry 6rq7 (GD)
Crystallization and data collection			
Stock solution	5 mg ml^−1^ afamin in 20 m*M* HEPES pH 7.5, 150 m*M* NaCl		5 mg ml^−1^ afamin in 20 m*M* HEPES pH 7.5, 150 m*M* NaCl, incubated with 10 m*M* Gd-DO3A
Crystallization conditions	1.5 µl afamin stock + 1.5 µl precipitant in hanging-drop vapour diffusion		200 nl afamin stock + 200 nl precipitant in hanging-drop vapour diffusion (EMBL HTX)
	Precipitant: 30% PEG 1K, 170 m*M* ammonium acetate, 3% 6-aminohexanoic acid		Precipitant: 23–28% PEG 4K, 0.2 *M* ammonium sulfate, 0.1 *M* ammonium acetate pH 5.5
Beamline	MASSIF-1, ESRF	MASSIF-1, ESRF	MASSIF-1, ESRF
Wavelength (Å)	0.966	0.966	0.966
ESRF data identification	https://doi.esrf.fr/10.15151/ESRF-DC-142893590	https://doi.esrf.fr/10.15151/ESRF-DC-142915526	https://doi.esrf.fr/10.15151/ESRF-DC-186857652 (CD024584_B04-3_2_1)
Crystal dimensions (mm)	0.430 × 0.044 × 0.191	0.287 × 0.043 × 0.178	0.071 × 0.074 × 0.081
Space group	*P*2_1_ [No. 4]	*P*2_1_2_1_2 [No. 18]	*P*2_1_2_1_2 [No. 18]
*a*, *b*, *c* (Å)	50.92, 112.78, 109.23	109.87, 113.23, 48.80	103.35, 109.73, 48.38
α, β, γ (°)	90.0, 93.4, 90.0	90.0, 90.0, 90.0	90.0, 90.0, 90.0
Unit-cell volume (Å^3^)	625561	607023	548658
Solvent fraction	0.470	0.453	0.398
*V*_M_ (Å^3^ Da^−1^)	2.32	2.25	2.05
Wilson *B* factor (Å^2^)	42.3	28.3	59.1
ISa	24.4	18.1	10.6
Resolution (Å)	49.09–2.08 (2.16–2.08)	78.86–1.90 (1.94–1.90)	75.24–2.70 (3.02–2.70)
Anisotropic (Å)	—	—	2.69, 3.09, 3.50
Completeness (%)	90.1 (76.8)	98.9 (95.8)	61.3 (11.0)
Anisotropic (%)	—	—	88.3 (61.5)
Observed reflections	194144 (15478)	170754 (9971)	45081 (1021)
Average multiplicity	2.93 (2.85)	3.55 (3.36)	4.7 (2.1)
〈*I*/σ(*I*)〉	13.0 (1.4)	14.9 (2.0)	5.6 (1.7)
*R*_meas_ †	0.054 (1.00)	0.057 (1.03)	0.202 (0.682)
*R*_merge_ †	0.045 (0.821)	0.042 (0.843)	0.180 (0.539)
CC_1/2_ ‡	0.999 (0.605)	0.999 (0.782)	0.993 (0.800)
Refinement			
Resolution (Å)	109.04–2.09 (2.14–2.09)	78.85–1.90 (1.95–1.90)	75.24–2.69 (2.76–2.69)
*R*_work_, *R*_free_ (5% set)	0.238 (0.380), 0.200 (0.370)	0.214 (0.400), 0.182 (0.333)	0.262 (0.345), 0.335 (0.387)
Coordinate errors (free, σ_A_, DPI) (Å)	0.204, 0.184, 0.274	0.143, 0.117, 0.167	0.719, 0.556, 0.719
*F*_o_ versus *F*_c_ correlation, free	0.960, 0.939	0.961, 0.949	0.885, 0.802
All refined non-H 〈*B*〉 (Å^2^)	61.7	44.0	56.2
TLS groups	4 + 4 (3 HSA + C-tag)	4 (3 HSA + C-tag)	0
PEG fragments	0	15	0
Missing residues	28 [4.8%], 33 [0.56%]	11 [1.9%]	107 [18%]
Geometry			
R.m.s.d., bond lengths (Å)	0.010	0.008	0.002
R.m.s.d., angles (°)	1.34	1.26	1.20
Ramachandran statistics			
Preferred§	1035 [96.4%]	488 [95.3%]	428 [91.5%]
Allowed§	30 [2.8%]	22 [4.3%]	33 [7.0%]
Outliers§	9 [0.8%]	2 [0.4%]	7 [1.5%]

† As defined in Einspahr & Weiss (2012 ▸).

‡ CC_1/2_ is Pearson’s correlation coefficient between two randomly assigned data sets, each derived by averaging half of the observations for a given reflection, as defined in Karplus & Diederichs (2012 ▸).

§ Determined using the Ramachandran plot boundaries in *Coot* (Emsley *et al.*, 2010 ▸)
